# Associations Between Cancer Risk Perceptions, Self-Efficacy, and Health Behaviors by BMI Category and Race and Ethnicity

**DOI:** 10.1007/s12529-023-10225-7

**Published:** 2023-11-21

**Authors:** Adaora Ezeani, Brianna Boggan, Lorenzo N. Hopper, Olga M. Herren, Tanya Agurs-Collins

**Affiliations:** 1 National Cancer Institute, Rockville, MD 20850 USA; 2 Department of Public Health Sciences, College of Health and Human Services, The University of North Carolina at Charlotte, Charlotte, USA; 3 National Institute on Minority Health and Health Disparities, Bethesda, MD 20982 USA

**Keywords:** Cancer prevention, Obesity, Obesity-related cancer, Cancer risk beliefs, Health behaviors

## Abstract

**Background:**

Cancer risk perceptions and high health-related self-efficacy may impact health behaviors and reduce risk of developing obesity-related cancers. The purpose of this study was to examine whether there are differences in associations among cancer risk perceptions, health-related self-efficacy, and health behaviors between people with healthy weight (PwHW) and people with overweight or obesity (PwO/O), and whether these associations vary by race and ethnicity.

**Method:**

Data from the Health Information National Trends Survey (HINTS) 5 Cycles 2 and 3 were used. Data from 6944 adults were analyzed using multivariate logistic regression to assess associations among study variables.

**Results:**

PwO/O who believed there are too many cancer prevention recommendations had lower log odds of meeting guidelines for strength training (*β* − 0.28; CI − 0.53 to − 0.04; *p* < 0.05) compared to PwHW. PwO/O who believed that obesity influences cancer risk were associated with low sedentary behavior (*β* 0.29; CI 0.05–0.54; *p* < 0.05) compared to PwHW. NHB PwO/O who held fatalistic beliefs and reported high self-efficacy ordered less food (e.g., fewer food items, foods with less calories, or smaller food sizes) compared to NHB Pw/HW (*p* < 0.05).

**Conclusion:**

Health behavior differences in PwHW and PwO/O may be associated with differences in cancer risk beliefs and health-related self-efficacy. Findings support the need for further research considering BMI and race and ethnicity in obesity-related cancer prevention and control.

## Introduction

Excess body weight, a preventable risk factor for serious diseases including type II diabetes, cardiovascular disease, and cancer, has significantly increased in the past two decades [1]. It is also an important factor in mortality, attributing to 15–20% of all cancer deaths in the USA [2]. The chronic disease burden related to adiposity can be mitigated by increasing the prevalence of health-promoting behaviors, physical activity, and nutrition. Recent studies have indicated that meeting physical activity guidelines is associated with decreased cancer incidence and mortality. The 2018 World Cancer Research Fund/American Institute for Cancer Research (WCRF/AICR) and 2018 Physical Activity Guidelines for Americans (PAGA) Committee reports concluded that there is evidence of reduced cancer risk with increased physical activity and increased risk with high levels of sedentary time, respectively [3]. There is also an inordinate amount of evidence that associates nutrition with cancer risk. Awareness of nutritional recommendations, such as dietary fiber intake and red and processed meat consumption, could reduce the risk of specific cancers by up to 30% [4, 5].

How individuals perceive their risk for developing cancer is a crucial variable when engaging in health promotion efforts intended to reduce the risk of developing obesity-related cancers. Several decision-making models allude to the importance of psychological factors enabling engagement in health-promoting behaviors [6, 7]. For example, cancer fatalism, or the belief that cancer diagnosis is predetermined or inevitable, is adversely associated with cancer risk-modifying behaviors, while self-efficacy, or confidence in one’s ability to care for their health, has been positively associated with health-promoting behaviors such as physical activity and healthy eating [8]. However, few studies have explored how weight status may influence individual’s perceptions of cancer risk, which can affect commitment to healthy eating and physical activity. Individuals at higher weight may have complex beliefs, such as internalized stigma, that may affect cancer risk and health behaviors. Furthermore, it is important to understand the relationship between cancer risk perceptions, health-related self-efficacy, and health behaviors by body mass index (BMI) status and differences between racial and ethnic groups. Disparities in the prevalence of obesity exists across racial and ethnic groups influenced by social determinants of health, including accessibility to healthy foods and availability of time and safe spaces for physical activity, creating barriers that impact the ability to maintain a healthy weight [7, 10].

Examining how these psychological determinants influence obesity-related health behaviors and how they differ by BMI status, race, and ethnicity will better inform obesity-related cancer prevention and control programs [9]. Therefore, this study aims to examine differences in associations between cancer risk perception, health-related self-efficacy, and health behaviors by BMI status (PwO/O versus PwHW). In addition, these associations will be assessed by race and ethnicity to explore whether differences in these relationships may contribute to disparities in risk of obesity.

## Method

### Study Population and Design

This study analyzed data from the Health Information National Trends Survey (HINTS) 5 Cycles 2 and 3*,* a nationally representative survey of US adults that regularly collects data about the public’s knowledge of, attitudes towards, and use of cancer- and health-related information [10]. The dataset consisted of 8773 participants, adults 20 years and older, recruited between January and May 2018 and January and May 2019. Only biologically plausible heights and weights were included in the analyses (> 70 lb and < 700 lb; > 46 in. and < 84 in.) (*n* = 5) [11]. Participants were excluded if they had a prior cancer diagnosis (*n* = 1377) or a BMI lower than 18.5 (lbs/in.^2^) (*n* = 109), resulting in a total of 6944 participants. All study variables were cognitively tested before study implementation to ensure participants were able to understand and complete the instruments [10]. Ethical approval and participant consent was not required as this study involved the use of publicly available de-identified data.

### Descriptive Variables

Sociodemographic variables measured included self-reported gender (man or woman), age (18–49, 50–69, and 70 +), race and ethnicity (non-Hispanic White [NHW], non-Hispanic Black [NHB], and Hispanic/Latino), educational attainment, (high school or less, some college, and college graduate), annual household income (less than $34,999, $35,000–$74,999, and $75,000 +), marital status (married/living with partner, divorced/widow/separated/and single), general health status (excellent/very good, good, and fair/poor), and family history of cancer. Self-reported height and weight were used to calculate BMI (lbs/in.^2^). Participant’s BMI was then categorized into healthy weight (PwHW) (18.5–24.9 lbs/in.^2^) or overweight/obese (PwO/O) (> 25.0 lbs/in.^2^) to form comparison groups.

### Cancer Risk Perceptions and Health-Related Self-Efficacy

#### Cancer Risk Perceptions

To assess cancer fatalistic beliefs, participants were asked their level of agreement on a 4-point Likert scale on the following HINTS survey items: (1) “*It seems like everything causes cancer*,” (“everything causes cancer”) (2) “*There is not much you can do to lower your chances of getting cancer*,” (“prevention is not possible”) and (3) “*There are so many different recommendations about preventing cancer*” (“too many recommendations”). Responses were dichotomized into “agree” (“strongly agree” and “somewhat agree”) and “disagree” (“strongly disagree” and “somewhat disagree”). The three items measure distinct dimensions of cancer fatalistic beliefs, which are known to show predictive capability for cancer preventive health behaviors. They were analyzed separately due to low positive correlations (*r*_1, 2_ = 0.21, *r*_1,3_ = 0.27, *r*_2,3_ = 0.27; all *p* < 0.001). Cancer risk beliefs related to obesity were assessed with the item: “*How much can obesity influence whether or not a person will develop cancer?*” (“obesity influences cancer”). Responses were dichotomized into “a lot or a little” (“a lot,” and “a little”) and “not at all” (“not at all,” and “don’t know”). These items were pretested with cognitive interviews and included in a national pilot test to ensure content validity [10].

#### Health-Related Self-Efficacy

Health-related self-efficacy was measured by a survey item asking participants to rate their confidence in their ability to care for their health (“*Overall, how confident are you about your ability to take good care of your health*?”). Five-point Likert responses were categorized into “confident” (“completely confident”, “very confident”, and “somewhat confident”) and “not confident” (“a little confident” and “not confident at all”).

### Health Behaviors

#### Physical Activity

Participants responded to the following physical activity–related items: “*How many days do you do any physical activity or exercise of at least moderate intensity, such as brisk walking, bicycling at a regular pace, swimming at a regular pace, and heavy gardening?*” and “*On the days that you do any physical activity or exercise of at least moderate intensity, how long are you typically doing these activities*?” Responses were used to calculate weekly minutes of moderate physical activity; then results were dichotomized into “ ≥ 150 min per week” or “ < 150 min per week” based on adult physical activity recommendations per US Department of Health and Human Services guidelines [12]. Strength training was measured using the following item: “*How many days do you do leisure-time physical activities specifically designed to strengthen your muscles such as lifting weights or circuit training (do not include cardio exercises such as walking, biking, or swimming)?*”. Responses were dichotomized into “ ≥ 2 days per week” or “ < 2 days per week,” according to resistance training recommendations for adults based on US Department of Health and Human Services guidelines [12].

#### Sedentary Behavior

Sedentary behavior was assessed using the following item: “*During the past 7 days, how much time did you spend sitting on a typical day at home or at work*”. Responses were dichotomized into “ ≥ 6 h per day” or “ < 6 h per day.” Responses were dichotomized according to previous research identifying 6 h spent being sedentary as elevating the risk for adverse health outcomes [13, 14].

#### Food Ordering Behavior

After responding “yes” to the survey item, “*Thinking about the last time you ordered food in a fast food or sit-down restaurant, did you notice calorie information listed next to the food on the menu or menu board*?,” participants were asked the following survey items to measure food ordering behavior: “*How did the calorie information change what you were thinking of ordering*?” Participants answered “yes” or “no” to the following: “*I ordered something with fewer calories*”, “*I ordered something with more calories*”, “*I ordered fewer items*”, “*I ordered more items*”, “*I ordered smaller sizes*”, and “*I ordered something in larger sizes*”. To better capture food behavior, responses were operationalized as five distinct categories based on previous research by Rising et al. [15]: (1) No difference in food ordering behavior (“no” to all six response options, (2) Ordering less in one way *(*“yes” to “*fewer items*,” “*fewer calories*,” or “*smaller sizes*” and “no” to “*more items*,” “*more calories*” and “*larger sizes*”), (3) Ordering less in two or more ways (“yes” to “*fewer items*” and “*fewer calories*” or “*fewer items*” and “*smaller sizes*,” or “*fewer calories*” and “*smaller sizes*” and “no” to “*more items*,” “*more calories*,” and “*larger sizes*”), (4) Ordering less and ordering more (“yes” to “*fewer items*,” “*fewer calories*,” or “*smaller sizes*” and “*yes*” to “*more items*,” “*more calories*” or “*larger sizes*”), and (5) Ordering more and not ordering less (“*yes*” to “*more items*,” “*more calories*” or “*larger sizes*” and “no” to “*fewer items*,” “*fewer calories*,” or “*smaller sizes*”). These measures were only available to respondents who reported noticing menu calorie information.

### Statistical Analysis

Descriptive statistics and chi-square tests of independence were performed to compare sample characteristics by BMI group. Binomial logistic regression was used to examine associations between independent variables, cancer risk perceptions, and health-related self-efficacy, with dependent variables, physical activity, sedentary behavior, and strength training, comparing PwO/O with PwHW. Then, multinomial logistic regression analysis examined associations between independent variables and food ordering behavior, with no difference in food ordering behavior category as reference. Regression models were adjusted for gender, age, race and ethnicity, educational attainment, income, marital status, and general health status. Subsequently, data were stratified and analyses were conducted to examine whether patterns differed by racial and ethnic groups. Analyses were completed using Stata/SE (version 17.0), applying survey replicate weights and variance estimation methods to ensure valid population-level inferences [16, 17]. All analyses employed a two-sided *p*-value < 0.05.

## Results

The sociodemographic characteristics of participants are presented in Table 1. The total sample in current analyses included data from 6944 participants, of which approximately 30% reported a healthy BMI status, 50% were men, 69% were NHW, 40% reported completing some college education, and 40.5% had an annual household income greater than $75,000. The age group most represented in the current sample was 20–49 (51.5%). More than half of the participants were married/living with a partner (55.2%) and self-reported excellent/very good health (51%). Women accounted for 55% of the PwHW and men accounted for 52.2% of PwO/O (*p* = 0.002). The youngest age group, 20–49 years old, accounted for 57.8% of PwHW and 48.9% of PwO/O (*p* < 0.001). Participants who attained a college degree accounted for 40.4% of the PwHW and those with some college education accounted for 41.8% of PwO/O (*p* < 0.001). Participants with income greater than $75,000 (44.9%) and self-reported excellent/very good health (68.4%) had a higher representation of PwHW compared to PwO/O (*p* = 0.02; *p* < 0.001).

**Table 1:** Sociodemographic characteristics (%)^a^ of participants by healthy and overweight/obese BMI^b^

**Variables**	**Healthy BMI** (*n* = 2098)	**Overweight/obese BMI** (*n* = 4846)	**Total sample** (*n* = 6944)
Gender^**^			
Male	45.0	52.2	50.0
Female	55.0	47.8	50.0
Age (years)^***^			
20–49	57.8	48.9	51.5
50–64	25.0	34.5	31.6
65 +	17.3	16.6	16.8
Race and ethnicity^***^			
Non-Hispanic White	76.3	66.61	69.0
Non-Hispanic Black	8.24	14.5	12.7
Hispanic/Latino	15.4	19.4	18.3
Education^***^			
High school or less	23.5	32.7	29.9
Some college	36.1	41.8	40.0
College graduate	40.4	25.6	30.1
Annual household income^*^			
Less than $34,999	28.0	29.1	28.8
$35,000–$74,999	27.1	32.2	30.7
$75,000 +	44.9	38.7	40.5
**Marital status**			
Married/living with partner	52.3	56.4	55.2
Divorced/widowed/separated	14.5	15.1	14.9
Single	33.1	28.5	29.9
General health status^***^			
Excellent/very good	68.4	43.5	51.0
Good	23.6	39.3	34.6
Fair/poor	8.0	17.1	14.4
**Family history of cancer**			
Yes	74.4	75.2	74.9

^a^Weighted percentages

^b^*BMI*, body mass index

^*^p < 0.05; ^**^p < 0.01; ^***^p < 0.001

### Associations of Cancer Risk Beliefs and Health-Related Self-Efficacy with Health Behaviors

Table 2 presents results from multivariate logistic regression analyses assessing the main effects of cancer risk perceptions and health-related self-efficacy on health behaviors. PwO/O who believed there are “too many recommendations” about cancer prevention had lower log odds of reporting participation in recommended strength training guidelines (*β* − 0.28; CI − 0.53 to − 0.04) compared to PwHW. PwO/O who agreed that “obesity influences cancer” had lower log odds of reporting low sedentary behavior (*β* 0.29; CI 0.05–0.54) compared to PwHW. In addition, PwO/O who agreed that “everything causes cancer”, “obesity influences cancer”, and were confident in their ability to take care of their health had higher log odds of noticing calorie information on menus compared to PwHW (Table 3).

**Table 2:** Associations (β) between cancer risk perceptions and health-related self-efficacy with physical activity recommendations among PwO/O compared to PwHW

	Strength training	Low sedentary activity	Moderate exercise
Everything causes cancer	− 0.15 [− 0.42,0.12]	0.12 [− 0.14,0.39]	− 0.25 [− 0.53,0.04]
Prevention is not possible	− 0.08 [− 0.53,0.37]	0.19 [− 0.27,0.65]	− 0.29 [− 0.82,0.23]
Too many recommendations	− 0.28^*^ **[**− 0.53, − 0.04]	0.11 [− 0.17,0.40]	− 0.26 [− 0.55,0.03]
Obesity influences cancer risk	− 0.22 [− 0.48,0.04]	0.29^*^ [0.05,0.54]	− 0.06 [− 0.34,0.21]
Own ability to take care of health	− 0.18 [− 0.40,0.03]	0.14 [− 0.09,0.37]	− 0.16 [− 0.41,0.08]

Adjusted by gender, age, race and ethnicity, education, income, marital status, and general health status

^*^p < 0.05; ^**^p < 0.01; ^***^p < 0.001

**Table 3:** Associations (*β*) between cancer risk perceptions and health-related self-efficacy with food ordering behavior among PwO/O compared to PwHW

	No difference	Order less in one way^a^	Order less in ≥ 2 ways	Order less/order more	Order more only
Everything causes cancer	Ref.	0.29 [− 0.26,0.85]	0.20 [− 0.34,0.74]	0.29 [− 0.85,1.44]	− 0.02 [− 1.07,1.03]
Prevention is not possible	Ref.	0.40 [− 0.45,1.26]	0.76 [− 0.01,1.53]	1.89 [− 0.15,3.93]	0.64 [− 1.26,2.54]
Too many recommendations	Ref.	0.01 [− 0.46,0.48]	− 0.03 [− 0.49,0.42]	− 0.09 [− 1.53,1.34]	− 0.16 [− 1.21,0.90]
Obesity influences cancer risk	Ref.	0.27 [− 0.26,0.80]	0.28 [− 0.21,0.77]	− 0.73 [− 1.82,0.36]	− 0.04 [− 1.13,1.06]
Own ability to take care of health	Ref.	0.07 [− 0.36,0.51]	0.13 [− 0.30,0.57]	− 0.04 [− 1.28,1.20]	− 0.04 [− 1.04,0.96]

Adjusted by gender, age, race and ethnicity, education, income, marital status, and general health status

^a^Fewer food items, foods with less calories, or smaller food sizes

^*^p < 0.05; ^**^p < 0.01; ^***^p < 0.001

### Associations Between Cancer Risk Beliefs, and Health-Related Self-Efficacy and Health Behaviors by Race and Ethnicity

NHW PwO/O who believe there are “too many recommendations” had lower log odds of participating in more than 150 min of moderate exercise per week (*β* − 0.35, CI − 0.70 to 0.00) (Tables 4 and 5). No statistically significant findings for physical activity and sedentary behavior were present among NHB (Tables 6 and 7) and Hispanic/Latino groups (Tables 8 and 9).

**Table 4:** Associations (*β*) between cancer risk perceptions and health-related self-efficacy with physical activity recommendations among NHW PwO/O compared to NHW PwHW

	Strength training	Low sedentary activity	Moderate exercise
Everything causes cancer	− 0.23 [− 0.56,0.10]	0.14 [− 0.19,0.47]	− 0.30 [− 0.65,0.05]
Prevention is not possible	− 0.02 [− 0.63,0.59]	0.43 [− 0.16,1.01]	− 0.26 [− 0.92,0.41]
Too many recommendations	− 0.30 [− 0.61,0.01]	0.13 [− 0.22,0.49]	− 0.35^*^ **[**− 0.70, − 0.00]
Obesity influences cancer risk	− 0.22 [− 0.51,0.06]	0.25 [− 0.05,0.55]	− 0.08 [− 0.42,0.25]
Own ability to take care of health	− 0.23 [− 0.48,0.03]	0.14 [− 0.15,0.43]	− 0.21 [− 0.50,0.09]

Adjusted by gender, age, race and ethnicity, education, income, marital status, and general health status

^*^p < 0.05; ^**^p < 0.01; ^***^p < 0.001

**Table 5:** Associations (*β*) between cancer risk perceptions and health-related self-efficacy with food ordering behavior among NHW PwO/O compared to NHW PwHW

	No difference	Order less in one way^a^	Order less in ≥ 2 ways	Order less/order more	Order more only
Everything causes cancer	Ref	0.19 [− 0.44,0.82]	0.19 [− 0.38,0.77]	0.84 [− 1.29,2.96]	− 0.25 [− 1.70,1.20]
Prevention is not possible	Ref	0.39 [− 0.44,1.23]	0.82^*^ [0.01,1.63]	2.87 [− 0.49,6.24]	− 0.47 [− 3.11,2.17]
Too many recommendations	Ref	0.00 [− 0.52,0.52]	0.02 [− 0.46,0.49]	0.45 [− 1.66,2.57]	− 0.76 [− 2.29,0.77]
Obesity influence cancer risk	Ref	0.34 [− 0.22,0.90]	0.27 [− 0.24,0.79]	− 0.80 [− 2.10,0.51]	− 0.19 [− 1.75,1.36]
Own ability to take care of health	Ref	0.08 [− 0.39,0.55]	0.15 [− 0.32,0.62]	0.18 [− 1.62,1.98]	− 0.71 [− 2.03,0.61]

Adjusted by gender, age, race and ethnicity, education, income, marital status, and general health status

^a^Fewer food items, foods with less calories, or smaller food sizes

^*^p < 0.05; ^**^p < 0.01; ^***^p < 0.001

**Table 6:** Associations (*β*) between cancer risk perceptions and health-related self-efficacy with physical activity recommendations among NHB PwO/O compared to NHB PwHW

	Strength training	Low sedentary activity	Moderate exercise
Everything causes cancer	0.58 [− 0.20,1.37]	− 0.28 [− 1.14,0.58]	− 0.12 [− 0.92,0.68]
Prevention is not possible	0.40 [− 0.58,1.39]	− 0.79 [− 1.90,0.31]	0.15 [− 0.98,1.29]
Too many recommendations	0.48 [− 0.32,1.27]	− 0.40 [− 1.16,0.36]	0.15 [− 0.68,0.98]
Obesity influences cancer risk	0.03 [− 0.76,0.82]	0.02 [− 0.85,0.90]	− 0.27 [− 1.04,0.50]
Own ability to take care of health	0.38 [− 0.34,1.09]	− 0.18 [− 0.85,0.48]	0.45 [− 0.23,1.12]

Adjusted by gender, age, race and ethnicity, education, income, marital status, and general health status

^*^p < 0.05; ^**^p < 0.01; ^***^p < 0.001

**Table 7:** Associations (*β*) between cancer risk perceptions and health-related self-efficacy with food ordering behavior among NHB PwO/O compared to NHB PwHW

	No difference	Order less in one way^a^	Order less in ≥ 2 ways	Order less/order more	Order more only
Everything causes cancer	Ref	2.77^**^ [0.99,4.54]	1.68 [− 0.04,3.40]	0.57 [− 1.94,3.08]	0.79 [− 2.25,3.83]
Prevention is not possible	Ref	1.10 [− 1.19,3.39]	2.72^*^ [0.64,4.81]	0.58 [− 1.87,3.03]	− 2.21 [− 4.65,0.23]
Too many recommendations	Ref	2.15^**^ [0.72,3.59]	1.54 [− 0.02,3.09]	0.48 [− 1.66,2.62]	0.65 [− 2.11,3.42]
Obesity influences cancer risk	Ref	1.43 [− 0.30,3.17]	1.59^*^ [0.13,3.06]	− 0.92 [− 2.45,0.62]	− 0.32 [− 2.12,1.48]
Own ability to take care of health	Ref	1.82^**^ [0.64,3.00]	1.39^*^ [0.15,2.63]	0.91 [− 1.24,3.06]	0.95 [− 1.19,3.10]

^*^p < 0.05; ^**^p < 0.01; ^***^p < 0.001

**Table 8:** Associations (*β*) between cancer risk perceptions and health-related self-efficacy with physical activity recommendations among Hispanic PwO/O compared to Hispanic/Latino PwHW

	Strength training	Low sedentary activity	Moderate exercise
Everything causes cancer	0.07 [− 0.48,0.62]	− 0.01 [− 0.62,0.60]	0.27 [− 0.46,1.00]
Prevention is not possible	0.26 [− 0.61,1.14]	− 0.45 [− 1.26,0.36]	− 0.33 [− 1.28,0.61]
Too many recommendations	− 0.36 [− 0.91,0.18]	0.11 [− 0.50,0.73]	− 0.04 [− 0.75,0.68]
Obesity influences cancer risk	− 0.18 [− 0.74,0.39]	0.25 [− 0.42,0.93]	0.36 [− 0.33,1.05]
Own ability to take care of health	− 0.22 [− 0.69,0.25]	− 0.01 [− 0.53,0.51]	− 0.05 [− 0.64,0.54]

Adjusted by gender, age, race and ethnicity, education, income, marital status, and general health status

^*^p < 0.05; ^**^p < 0.01; ^***^p < 0.001

**Table 9:** Associations (*β*) between cancer risk perceptions and health-related self-efficacy with food ordering behavior among Hispanic/Latino PwO/O compared to Hispanic/Latino PwHW

	No difference	Order less in one way^a^	Order less in ≥ 2 ways	Order less/order more	Order more only
Everything causes cancer	Ref	1.72 [− 0.50,3.94]	0.15 [− 1.10,1.40]	− 0.63 [− 3.08,1.81]	1.06 [− 1.69,3.80]
Prevention is not possible	Ref	1.54 [− 1.26,4.34]	0.74 [− 0.88,2.35]	− 0.27 [− 3.39,2.86]	17.67 [− 1.32,36.65]
Too many recommendations	Ref	0.40 [− 1.81,2.62]	− 0.51 [− 1.94,0.92]	− 1.38 [− 4.28,1.52]	1.01 [− 2.04,4.07]
Obesity influence cancer risk	Ref	0.13 [− 1.63,1.88]	− 0.37 [− 1.92,1.19]	0.12 [− 2.61,2.86]	0.13 [− 2.62,2.88]
Own ability to take care of health	Ref	0.29 [− 1.16,1.75]	− 0.02 [− 1.07,1.02]	0.18 [− 2.44,2.80]	1.04 [− 1.52,3.61]

Adjusted by gender, age, race and ethnicity, education, income, marital status, and general health status

^a^Fewer food items, foods with less calories, or smaller food sizes

^*^p < 0.05; ^**^p < 0.01; ^***^p < 0.001

Regarding food ordering behavior, NHW PwO/O who agreed that “everything causes cancer”, “obesity influences cancer risk”, and reported high self-efficacy had higher log odds of noticing calorie information on menus compared to NHW PwHW (*β*_everything_ 0.37, CI 0.04–0.71; *β*_influence_ 0.39, CI 0.09–0.69; *β*_self-efficacy_ 0.37, CI 0.08–0.66). Of those who were aware of calorie information on menus, NHW PwO/O who believe “prevention is not possible” were more likely to order fewer food items (*β* 0.82, CI 0.01–1.63) compared to NHW PwHW (Table 5). For NHB, PwO/O who believed “everything causes cancer” and “too many recommendations” had higher log odds of ordering less food in one way (e.g., fewer food items, foods with less calories, or smaller food sizes), and those whose who believed “prevention not possible” had higher log odds of ordering less in 2 or more ways, compared to NHB PwHW (Table 7). In addition, NHB PwO/O who agreed “obesity influences cancer” had higher log odds of ordering less in two ways or more (*β* 1.59, CI 0.13–3.06) compared to NHB Pw/HW. NHB PwO/O who reported high self-efficacy had higher log odds of ordering less in one way (*β* 1.82, CI 0.64–3.00) or two ways or more (*β* 1.39, CI 0.15–2.63) compared to NHB Pw/HW. There were no significant associations for Hispanic/Latinos related to cancer-related perceptions and health-related self-efficacy with food ordering behavior.

## Discussion

This study aimed to explore differences in associations between cancer risk perceptions, health-related self-efficacy, and health behaviors among US PwO/O compared to PwHW. Overall, PwO/O who hold these cancer risk perceptions demonstrate a decreased association with physical activity but increased association with low sedentary behavior. However, statistical significance was apparent only in PwO/O who agreed there were “too many recommendations” about preventing cancer and decreased participation in strength training. Also, PwO/O who agreed “obesity influences cancer” had low sedentary behavior compared to their PwHW counterparts. These results suggest that PwO/O who hold fatalistic cancer prevention beliefs may be less engaged in adopting exercise or physical activity maintain or improve their health. Similar to previous studies, PwO/O who agreed with fatalistic beliefs were less likely than PwHW to cite exercise as a cancer preventive behavior [18, 19]. Most may opt to adopt other behaviors, such as reducing sedentary activity.

In contrast, high health-related self-efficacy negatively influenced weight-management related health behaviors across BMI status [19, 20]. Self-efficacy has been known to be a major motivating factor in developing intentions to exercise and maintain the practice for an extended period of time [21–23]. Conflicting results may be attributed to the tendency of PwO/O to overestimate their self-efficacy regarding exercise, likely due to insufficient knowledge of the self-regulatory demands of physical activity [23, 24]. Although unable to meet physical activity recommendations, PwO/O with high self-efficacy may participate in light or irregular physical activity [24]. Greater emphasis should be placed on improving public awareness and understanding of the associations among obesity, cancer, and the importance of both eating behaviors and adequate physical activity, such that, even in the presence of conflicting or strong beliefs on these topics (i.e., “cancer prevention is not possible”), there is an impetus to reduce cancer risk by adopting healthy behaviors.

Significant racial and ethnic disparities in the prevalence of obesity [25] warrant stratifying analyses to develop a better understanding as to whether these group-based differences in the associations among cancer risk perceptions, health-related self-efficacy, and health behaviors contribute to these disparities. Among NHW, PwO/O who agreed cancer “prevention is not possible” demonstrated higher odds of ordering less food (e.g., fewer food items, foods with less calories, or smaller food sizes) compared to PwHw. NHW with obesity were more conscientious of calorie information and practice healthier eating habits, and despite their fatalistic belief, NHW PwO/O made a deliberate effort to manage weight through dietary intake [26]. Greater significance between cancer risk perceptions, self-efficacy, and food ordering behavior was found among NHB. Compared to NHB PwHW, NHB PwO/O, who held cancer fatalistic beliefs, agreed “obesity influences cancer,” and reported high self-efficacy had healthy food ordering behavior, such as ordering smaller food sizes, fewer items, and/or foods with less calories. Despite the pessimistic view about cancer prevention, dietary modification remains a significant and prevailing preventive health behavior, particularly among NHB PwO/O. For some PwO/O, there are significant barriers to regular exercise, such as lack of time and motivation, poor body image, poor confidence, and lack of immediate rewards [27]. Therefore, when making lifestyle changes to manage weight, this population may find it more feasible modifying food ordering behavior than increasing physical activity compared to participants with PwHW when making lifestyle changes to manage weight, as these changes require less time, energy, and resource commitment. In addition, there are lower rates of physical activity among racial and ethnic minority populations [28], and it may be easier to restrict calories with small adjustments in portion sizes, fewer items, or purchasing an item with fewer calories than participating in regular physical activity. Also, environmental and cultural context may play a large role in the strong preference for healthy eating present among NHB PwO/O compared to exercise. Social determinants of health beyond individual-level influences are more likely to impact NHB, limiting their lifestyle choices, which may be reflected in the limited health behavior outcomes observed in this study. In general, NHBs are disproportionately susceptible to social determinants of health, such as increased exposure to poor quality, decreased affordability of healthy foods, low access to safe spaces for physical activity, and less time and resources for physical activity engagement [27, 29].

Despite the added challenges among NHB, our findings suggest the importance of informing populations that experience obesity and how to navigate complex barriers and importance of increased physical activity, reduced sedentary behavior, and a healthy diet. Counseling with professionals, such as physicians or trusted community health educators, may help influence health beliefs and health behaviors among PwO/O. However, these intervention strategies and resources should be made widely available to create awareness and improve health behaviors to prevent obesity. It is also necessary to develop and implement multi-level interventions based on contextual factors, policy changes, community investment programs, and incorporate social support to increase the likelihood of long-term behavioral changes in these under-resourced populations. For example, policy changes mandating the inclusion of nutritional facts on food labels accompanied with information regarding the link between obesity and cancer can inform health beliefs and the adoption of healthy behaviors. Without these multi-level supports, the responsibility for health is disproportionately placed on these marginalized individuals, for whom structural racism has contributed to further disparities largely influenced by social conditions and limited options for healthy living rather than personal choice [30].

## Limitations

The current study was mostly male, NHW, and of higher socioeconomic status, which is not representative of all segments of the US population. However analyses examining differences by racial and ethnic subgroups can inform future research that focused on improving health in these populations. Additionally, several variables that may influence engagement in healthy lifestyle patterns, including aspects of the social determinants of health, such as food insecurity, and dietary information, such as weight management or 24-h diet recalls, are not available in the datasets to fully ascertain overall dietary patterns and predictors of food ordering behavior. Regarding cancer risk perception variables, possible unmeasured confounders include faith and religion. The self-efficacy measure was derived from a single survey item and may not be robust to impact specific health self-management tasks. In addition, responses to the self-efficacy question may be influenced by recall and social desirability bias. However, other studies have used this measure of self-efficacy and found good correlation with positive health behaviors [31–33]. The cross-sectional design of this study also limits the ability to define the temporal sequence of these associations, particularly how the interplay of BMI status, cancer risk perceptions, and health-related self-efficacy influence health behaviors that impact cancer risk. Additionally, potential recall bias from self-reported responses such as height and weight may overestimate or underestimate BMI status. However, the robust sample size may help to address this source of potential error. This study has several strengths, including the use of a large weighted sample that is representative of and generalizable to the US population, and is a resource for future studies focused on psychological factors and obesity-related behaviors.

## Conclusion

Cancer risk beliefs, health-related self-efficacy, and BMI status are hypothesized as important and understudied contributors to health behaviors related to cancer risk. Thus, they are important when developing cancer prevention and intervention programs. This study highlighted differences in cancer risk beliefs and health-related self-efficacy among individuals by BMI status (PwHW versus PwO/O) on health behaviors in a healthy sample of adults. Associations among cancer risk beliefs, health-related self-efficacy, and health behaviors with a consideration of how BMI status and race and ethnicity influence these relationships require further research. The inclusion of higher-level social determinants of health will help to inform development of interventions among minoritized sub-groups that struggle to adopt healthy lifestyle behaviors. Future prevention and intervention strategies should pay special attention to the beliefs and psychological processes that impact health behaviors among PwO/O as well as racial and ethnic groups, for example, identifying possible causal relationships or latent constructs between each cancer risk perception, health-related self-efficacy, and sociodemographic variables across BMI categories. In addition, exploring a wider array of factors such as health information technology, socio-economic determinants, cultural nuances, and psychological variables will undoubtedly enrich our understanding of the complex interplay in these domains. By investigating these additional dimensions, future research can advance our insights into effective strategies for promoting healthier behaviors and informed decision-making regarding cancer risks.

## Data Availability

Publicly available datasets were analyzed in this study. This data can be found here: https://hints.cancer.gov/data/default.aspx

## References

[1] Calle EE, Kaaks R. Overweight, obesity and cancer: epidemiological evidence and proposed mechanisms. Nat Rev Cancer. 2004;4(8):579–91. 15286738 10.1038/nrc1408

[2] Calle EE, et al. Overweight, obesity, and mortality from cancer in a prospectively studied cohort of US adults. N Engl J Med. 2003;348(17):1625–38. 12711737 10.1056/NEJMoa021423

[3] Friedenreich CM, Ryder-Burbidge C, McNeil J. Physical activity, obesity and sedentary behavior in cancer etiology: epidemiologic evidence and biologic mechanisms. Mol Oncol. 2021;15(3):790–800. 32741068 10.1002/1878-0261.12772 PMC7931121

[4] Grundy A, et al. Cancer incidence attributable to lifestyle and environmental factors in Alberta in 2012: summary of results. Canadian Medical Association Open Access Journal. 2017;5(3):E540–5. 10.9778/cmajo.20160045 PMC5621951 28687643

[5] Di Sebastiano KM, et al. Nutrition and cancer prevention: why is the evidence lost in translation? Adv Nutr. 2019;10(3):410–8. 30915435 10.1093/advances/nmy089 PMC6520044

[6] Bandura A. Self-efficacy mechanism in human agency. Am Psychol. 1982;37(2):122.

[7] Ajzen I. The theory of planned behavior. Organ Behav Hum Decis Process. 1991;50(2):179–211.

[8] Welch JD, Ellis EM. Sex differences in the association of perceived ambiguity, cancer fatalism, and health-related self-efficacy with fruit and vegetable consumption. J Health Commun. 2018;23(12):984–92. 30346886 10.1080/10810730.2018.1534905

[9] Valencia CI, et al. Interrogating patterns of cancer disparities by expanding the social determinants of health framework to include biological pathways of social experiences. Int J Environ Res Public Health. 2022;19(4):2455. 35206642 10.3390/ijerph19042455 PMC8872134

[10] Nelson DE, et al. The Health Information National Trends Survey (HINTS): development, design, and dissemination. J Health Commun. 2004;9(5):443–60; Discussion 81–4. 10.1080/10810730490504233 15513791

[11] Maguen S, et al. The relationship between body mass index and mental health among Iraq and Afghanistan veterans. J Gen Intern Med. 2013;28:563–70. 10.1007/s11606-013-2374-8 PMC3695271 23807066

[12] Committee PAGA. Physical Activity Guidelines Advisory Committee Report, 2018. Washington, DC: US Department of Health and Human Services; 2018.

[13] Stamatakis E, Bauman AE. The bold sedentary behavior recommendations in the new Canadian guidelines: are they evidence-based? Response to “Sedentary Behavior Research Network members support new Canadian 24-Hour Movement Guideline recommendations.” J Sport Health Sci. 2020;9(6):482–4. 33071163 10.1016/j.jshs.2020.09.013 PMC7749232

[14] Chomistek AK, et al. Relationship of sedentary behavior and physical activity to incident cardiovascular disease: results from the Women’s Health Initiative. J Am Coll Cardiol. 2013;61(23):2346–54. 23583242 10.1016/j.jacc.2013.03.031 PMC3676694

[15] Rising CJ, et al. US adults noticing and using menu calorie information: analysis of the National Cancer Institute’s Health Information National Trends Survey data. Prev Med. 2021;153:106824. 10.1016/j.ypmed.2021.106824 34600959

[16] Survey HINT. Analytics recommendations for HINTS 5, Cycle 2 Data. 2018; Available from: https://hints.cancer.gov/.

[17] Survey HINT. Overview of the HINTS cycle 3 survey and data analysis recommendations. 2021. Available from: https://www.cancer.gov/.

[18] Cameron M, et al. The role of overweight and obesity in perceived risk factors for cancer: implications for education. J Cancer Educ. 2010;25(4):506–11. 20217292 10.1007/s13187-010-0085-y

[19] Niederdeppe J, Levy AG. Fatalistic beliefs about cancer prevention and three prevention behaviors. Cancer Epidemiol Biomarkers Prev. 2007;16(5):998–1003. 17507628 10.1158/1055-9965.EPI-06-0608

[20] Fleary SA, et al. The relationship between health literacy, cancer prevention beliefs, and cancer prevention behaviors. J Cancer Educ. 2019;34(5):958–65. 30022378 10.1007/s13187-018-1400-2 PMC6339599

[21] Robertson MC, et al. Self-efficacy and physical activity in overweight and obese adults participating in a worksite weight loss intervention: multistate modeling of wearable device data. Cancer Epidemiol Biomarkers Prev. 2020;29(4):769–76. 31871110 10.1158/1055-9965.EPI-19-0907 PMC7125025

[22] Bauman AE, et al. Correlates of physical activity: why are some people physically active and others not? Lancet. 2012;380(9838):258–71. 22818938 10.1016/S0140-6736(12)60735-1

[23] McAuley E, Blissmer B. Self-efficacy determinants and consequences of physical activity. Exerc Sport Sci Rev. 2000;28(2):85–8. 10902091

[24] Buckley J. Exercise self-efficacy intervention in overweight and obese women. J Health Psychol. 2014;21(6):1074–84. 25145587 10.1177/1359105314545096

[25] National Health and Nutrition Examination Survey 2017–March 2020. Prepandemic data files development of files and prevalence estimates for selected health outcomes, in National Health Statistics Reports, S. National Center for Health, Editor. 2021. 10.15620/cdc:106273: Hyattsville, MD. 10.15620/cdc:106273 PMC11513744 39380201

[26] Christoph MJ, et al. Nutrition facts panels: who uses them, what do they use, and how does use relate to dietary intake? J Acad Nutr Diet. 2018;118(2):217–28. 29389508 10.1016/j.jand.2017.10.014 PMC5797995

[27] Stankevitz K, et al. Perceived barriers to healthy eating and physical activity among participants in a workplace obesity intervention. J Occup Environ Med 2017;59(8). 10.1097/JOM.0000000000001092 28692017

[28] Mendoza-Vasconez AS, et al. Promoting physical activity among underserved populations. Curr Sports Med Rep. 2016;15(4):290–7. 27399827 10.1249/JSR.0000000000000276 PMC5371027

[29] Bantham A, et al. Overcoming barriers to physical activity in underserved populations. Prog Cardiovasc Dis. 2021;64:64–71. 33159937 10.1016/j.pcad.2020.11.002

[30] Jones NL, et al. Cross-cutting themes to advance the science of minority health and health disparities. Am J Public Health. 2019;109(S1):S21-s24. 30699031 10.2105/AJPH.2019.304950 PMC6356138

[31] Lin AW, et al. Obesity status on associations between cancer-related beliefs and health behaviors in cancer survivors: implications for patient-clinician communication. Patient Educ Couns. 2021;104(8):2067–72. 33558109 10.1016/j.pec.2021.01.033 PMC8217116

[32] Sheeran P, et al. The impact of changing attitudes, norms, and self-efficacy on health-related intentions and behavior: a meta-analysis. Health Psychol. 2016;35(11):1178. 27280365 10.1037/hea0000387

[33] Schwarzer R, Fuchs R. Changing risk behaviors and adopting health behaviors: the role of self-efficacy beliefs. Self-Efficacy Chang Soc. 1995;259:288.

